# Factors for cognitive impairment in adult epileptic patients

**DOI:** 10.1002/brb3.1475

**Published:** 2019-12-21

**Authors:** Lei Wang, Shenggen Chen, Changyun Liu, Wanhui Lin, Huapin Huang

**Affiliations:** ^1^ Department of Neurology Union Hospital of Fujian Medical University Fuzhou China; ^2^ Department of Cardiac Surgery Union Hospital of Fujian Medical University Fuzhou China; ^3^ Department of Neurology and Geriatrics Union Hospital of Fujian Medical University Fuzhou China; ^4^ Fujian Key Laboratory of Molecular Neurology Fuzhou China

**Keywords:** cognitive impairment, epileptic patients, factors

## Abstract

**Objective:**

To analyze factors for cognitive impairment in epileptic patients.

**Methods:**

A total of 257 epileptic patients completed clinical memory scale (CMS) and 70 of them were further surveyed with mini‐mental state examination (MMSE), Montreal cognitive assessment (MoCA), digital symbol test (DSy), verbal fluency test, digit span test (DSp), Hamilton anxiety scale (HAMA) and Hamilton depression scale (HAMD). Monadic linear related analysis and multiple stepwise regression analysis were performed to evaluate the potential factors for cognitive impairment.

**Results:**

Educational level was correlated with scores of cognitive tests (*p* < .01), with a difference between the junior high school group and senior high school group (*p* < .01 or *p* < .05). Seizure frequency was negatively correlated with CMS scores (*p* < .01), with a difference between the group with a seizure frequency of less than once a year and other groups (*p* < .01). The kind of antiepileptic drugs (AEDs) was negatively correlated with CMS scores (*p* < .01), with a difference between the single‐drug group and the group taking more than two kinds of AEDs (*p* < .01). Depression scores were negatively correlated with MMSE, MoCA, DSy, DSp (*p* < .01 or *p* < .05), disease duration negatively with DSy (*p* < .01), and age negatively with MoCA (*p* < .05). Seizure type was correlated with DSy, and general seizure fared worse in the tests than other seizure types (*p* < .05).

**Conclusion:**

Educational level, seizure frequency, kinds of AEDs and depression can affect the cognitive function of epileptic patients. High educational level, good seizure control, single‐drug treatment and healthy psychological state are protective factors for cognitive function of epileptic patients.

## Graphical abstract

Educational level, seizure frequency, kinds of antiepileptic drugs, and depression can affect the cognitive function of epileptic patients. High educational level, good seizure control, single‐drug treatment, and healthy psychological state are protective factors for cognitive function of epileptic patients. These findings provide a new strategy for avoiding cognitive impairment in epileptic patients.

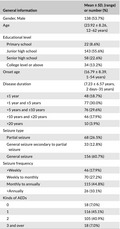

## 1 INTRODUCTION

Epilepsy is one of the most common neurological diseases, affecting about 65 million epileptic patients in the world (England, Liverman, Schultz, & Strawbridge, 2012; Jacobs & Jensen, 2012; Taylor & Baker, 2010), and 1 in 26 of the population may develop seizure at some points of their lives (Jacobs & Jensen, 2012). Epileptic patients are plighted with limited daily activities, social dysfunction, family conflicts and cognitive impairment (England et al., 2012). The latter has been reported in around 30%–40% of epileptic patients (Byard et al., 2007). Cognitive consequences in epilepsy are often described in the following domains: verbal memory, language, executive functions, and attention. However, most of the studies focus on one or two domains in a relatively small sample. For example, 31 studies have probed into the temporal lobe epilepsy with cognitive impairment in 357 patients and the meta‐analysis focuses on the social cognitive impairment (Bora & Meletti, 2016). Few studies focus on several domains at once. In the SANDA study, 155 newly diagnosed epileptic patients were enrolled and fared worse in 10 out of 16 items of cognitive function than the healthy group (Taylor et al., 2010). Another study enrolled 247 newly onset untreated epileptic patients, who showed 49.4% impairment in attention or executive function, 47.8% impairment in episodic memory, and 39.3% subjective deficits in attention and 35.2% subjective deficits in memory (Witt & Helmstaedter, 2012). Therefore, it is crucial to evaluate the cognitive function of epileptic patients and to seek interventions as soon as possible.

Cognition involves the brain's response to the objective world, ranging from simple perception of the environment and people themselves, attention, judgment, to the ability of performing complex mathematical calculation, language ability, memory, space ideation, executive function, etc. (Martin, Clare, Altgassen, Cameron, & Zehnder, 2011). Cognitive impairment in epileptic patients is often manifested in many cognitive domains, such as mild aprosexia or memory barriers, or falling of executive function, psychomotor speed, naming ability, visual–spatial ability and so on (Hermann et al., 2006; Piazzini, Turner, et al., 2006). These cognitive deteriorations can cause a significant decline in the life quality of epileptic patients and eventually cripple them (DiIorio et al., 2010). Few studies have investigated the influential factors for the epilepsy‐related cognitive impairment (Black et al., 2010; Gul & Ahmad, 2015; Henkin et al., 2005; Miller et al., 2016; Thompson & Duncan, 2005; Witt et al., 2014). However, most of these studies merely focused on one or a few potential factors and failed to consider possible distractions from other influential factors. Therefore, it is crucial to include more potential factors in the analysis and to rule out possible confounding interactions between factors.

At present, two main types of methods are currently employed for cognitive evaluation. One relies on cognitive function scales which are relatively subjective, including mini‐mental state examination (MMSE), Montreal cognitive assessment (MoCA), clinical memory scale (CMS), Wechsler adult intelligence scale, Rey auditory vocabulary learning test, and the Stroop color word interference test, etc. These scales are widely adopted in clinical cognitive evaluation due to their convenience and economy. The other employs neural electrophysiology and neuroimaging techniques which are relatively objective, such as positron emission tomography and functional magnetic resonance imaging.

Accordingly, the current study employed six neuropsychological scales, including CMS, MMSE, MoCA, digital symbol test (DSy), verbal fluency test (VFT), digit span test (DSp), and analyzed several potential factors to provide a comprehensive evaluation of the changes in the domain of memory, attention, calculating ability, executive function, visual space ability of the epileptic patients. The factors were classified into demographic factors, including gender, age, educational level; seizure‐related factors, including onset age, disease duration, seizure frequency, seizure types; therapeutic factor, including kinds of antiepileptic drugs (AEDs); and social psychological factors, including the state of anxiety and depression. This array of factors can provide a relatively comprehensive overview of the potential factors for cognitive impairment of the epileptic patients. Multiple stepwise regression analysis was performed to evaluate the cognitive performance of epileptic patients and to reduce the interference of other confounding factors.

## 2 SUBJECTS AND METHODS

### 2.1 Subjects

A total of 257 confirmed epileptic patients (according to the diagnosis of epilepsy in 2017, Fisher et al., 2017), 138 males and 119 females, were enrolled from the outpatient or inpatient neurology department of Union Hospital of Fujian Medical University from March 2015 to February 2016. Their demographic information, disease condition, and therapy received were summarized in Table 1. The inclusion criteria were as follows: (a) clinically confirmed typical seizure; (b) nonsymptomatic epilepsy, without CT or MRI indication of intracranial lesion; (c) cooperation with informed consent from the subjects or guardians; (d) an education level of primary school or above, with adequate literacy to read, understand, and respond to the scales correctly. The subjects were excluded from the study if they were afflicted with other psychoneurological illnesses, or had chronic history of alcoholism or drug abuse, or were recently on medications that may influence cognitive function and emotion (such as antidepressants and benzodiazepines drugs).

**Table 1:** The general information of epileptic patients (*n* = 257)

General information	Mean ± *SD*, (range) or number (%)
Gender, Male	138 (53.7%)
Age	(23.92 ± 8.26, 12–62 years)
Educational level	
Primary school	22 (8.6%)
Junior high school	143 (55.6%)
Senior high school	58 (22.6%)
College level or above	34 (13.2%)
Onset age	(16.79 ± 8.39, 1–54 years)
Disease duration	(7.23 ± 6.57 years, 2 days–31 years)
≤1 year	48 (18.7%)
>1 year and ≤5 years	77 (30.0%)
>5 years and ≤10 years	76 (29.6%)
>10 years and ≤20 years	46 (17.9%)
>20 years	10 (3.9%)
Seizure type	
Partial seizure	68 (26.5%)
General seizure secondary to partial seizure	33 (12.8%)
General seizure	156 (60.7%)
Seizure frequency	
>Weekly	46 (17.9%)
Weekly to monthly	70 (27.2%)
Monthly to annually	115 (44.8%)
<Annually	26 (10.1%)
Kinds of AEDs	
0	18 (7.0%)
1	116 (45.1%)
2	105 (40.9%)
3 and over	18 (7.0%)

Abbreviation: AEDs, antiepileptic drugs.

### 2.2 Data acquisition

General information and clinical data of patients were retrospectively analyzed including physical examination and other supportive examinations such as EEG, head CT or MRI. All examinations were not included in the treatment scheme and were conducted with the informed consent of the patients. So this study was approved by the ethics committee of the hospital.

### 2.3 Cognitive function and psychosocial tests

A total of 257 epileptic patients were surveyed by CMS, and 70 of them were simultaneously evaluated by MMSE, MoCA, DSy, VFT, DSp, Hamilton anxiety scale (HAMA), and Hamilton depression scale (HAMD).

#### 2.3.1 Clinical memory scale (CMS)

The scale, compiled by the Institute of Psychology of Chinese Social Sciences Academy, was composed of five sub‐items, including directed memory, associative learning, free image recall, meaningless graphic recognition, and associative memory of portrait characteristics. The subjects completed the scale in a quiet setting according to the instruction of the manual. The original scores of the five sub‐items were calculated and then converted into memory quotient according to the subjects’ age and educational level. Memory quotient was graded as follows: Super‐excellence = 130 points and above, excellence = 129–120 points, upper level = 119–110 points, moderate level = 109–90 points, lower level = 89–80 points, poor = 79–70 points, and very poor = below 69 points.

#### 2.3.2 Mini‐mental state examination (MMSE)

The examination was composed of 30 items (Folstein, Folstein, & McHugh, 1975), in which for each item, a correct answer is awarded one point, with a total of 30 points. This scale evaluates the subjects’ temporal and spatial orientation, verbal memory, attention and calculation, short‐term memory, object naming, retelling, reading comprehension, language comprehension, speech articulation, graphic drawing, etc. The normal reference value is related to the degree of education: illiteracy, more than 18 points: primary school educational level, more than 21 points; middle school educational level and above: more than 25 points. For patients with middle school educational level or above, mild abnormal was set at 21–24 points, moderate abnormal at 11–20 points, severe abnormal at <10 points.

#### 2.3.3 Montreal cognitive assessment (MoCA)

Montreal cognitive assessment is a rapid sensitive assessment tool for screening mild cognitive dysfunction (Nasreddine et al., 2005). The cognitive domain assessment includes attention, executive function, short‐term memory, verbal expression, abstract thinking, calculation, orientation, and visual–spatial ability. The total score was 30 points. A score of 26 points or over indicated a normal cognitive function. For people with <12 years of schooling, one point was added to the final total.

#### 2.3.4 Digital symbol test (DSy)

Digital symbol test is a sub‐test of Wechsler adult intelligence scale‐revised in China (WAIS‐RC), which evaluates attention, learning, operation speed, visual‐motion coordination and fine motion. The subjects were instructed to match a symbol with a number within 90 s according to the association between the number and the symbol. One point was awarded for each correct match. A score of 45 points or over indicated normal cognitive function.

#### 2.3.5 Verbal fluency test (VFT)

This test detects the ability of recalling certain kinds of things from memory and assesses instant verbal memory, spontaneous speech and thought organization. The subjects were asked to list as many names of fruits, animals, and vegetables as possible within 1 min, respectively, and the number of correct naming was recorded as the final score. The normal cognitive performance was rated against the education level: illiteracy, 16 points and over; primary school educational level, 21 points and over; and junior high school educational level and above, 26 points and over.

#### 2.3.6 Digit span test (DSp)

The test is also a sub‐test of WAIS‐RC, which mainly evaluates instant memory and attention by memorizing numbers in sequence and backward. The normal sequential memory was five digits and over, and the normal backward memory was four digits and over.

#### 2.3.7 Hamilton anxiety scale (HAMA)

This scale is composed of 14 items including the mood of anxiety, tension, fear, and insomnia. Scores of each item had five grades: those who were asymptomatic was zero; mild, moderate, severe, and very severe degrees were, respectively, determined as 1, 2, 3, and 4 points. The results were classified as follows: no anxiety, <7 points; potential anxiety, 7 points and over; anxiety, 14 points and over; obvious anxiety, 21 points and over; severe anxiety, 29 points and over.

#### 2.3.8 Hamilton depression scale (HAMD)

This scale is composed of 17 items including the mood of depression, guilt, sleeping difficulty, and suicide. Scores of each item had five grades: asymptom was zero; mild, moderate, severe, and very severe degrees were, respectively, determined as 1, 2, 3, and 4 points. The results were classified as follows: no depression, <7 points; potential depression, 7 points and over; depression, 17 points and over; severe depression, 25 points and over.

### 2.4 Grouping and analysis procedure

As the study was a retrospective design in nature and it was difficult to find a control group that matched the experimental group in terms of the age and education level, the cognitive function of epileptic patients was compared with the normal reference ranges.

Pearson correlation analysis or Spearman rank correlation analysis was used to analyze each potential factor affecting the cognitive function of epileptic patients, including age, educational level, onset age, disease duration, seizure frequency, seizure types, kinds of AEDs, anxiety, and depression.

According to the results of monadic linear related analysis above and clinical experience, multiple stepwise regression analysis was performed with age, educational level, disease duration, seizure frequency, seizure types, kinds of AEDs, degree of anxiety, and depression as independent variables and the scores of cognitive tests as dependent variables. The variables were coded as follows: for educational level, 1 meant primary school, 2 junior high school, 3 senior high school, and 4 college level or above; for seizure frequency, 1 meant less than once a year, 2 more than once a year and less than once a month, 3 more than once a month and less than once a week, and 4 more than once a week; for kinds of AEDs, 0 meant taking no drug, 1 taking one kind of drug, 2 two kinds of drugs, 3 three kinds of drugs or over; for seizure types, 1 meant partial seizure, 2 general seizure secondary to partial seizure, and 3 general seizure.

In order to investigate the effects of educational level, seizure frequency and kinds of AEDs on the cognitive function of the epileptic patients, they were grouped as follows:

In terms of their educational level, the epileptic patients were divided into four groups: the primary school group, junior high school group, senior high school group, and college level or above group. The cognitive function of the epileptic patients was compared across the groups.
In terms of the seizure frequencies, the epileptic patients were divided into four groups: the group with a frequency of less than once a year (Group A), the group with a frequency of more than once a year and less than once a month (Group B), the group of more than once a month and less than once a week (Group C), and the group of more than once a week (Group D). The cognitive function was compared across the above groups.
In terms of kinds of AEDs taken, the epileptic patients were divided into the following four groups: the group with no drugs (Group 0), the group taking one kind of drugs (Group 1), the group taking two kinds of drugs (Group 2), and the group taking three kinds of drugs or over (Group 3). The cognitive function was compared across the groups.

### 2.5 Statistical analysis

SPSS 20.0 software package for Windows was used for the statistical analysis of this study. Descriptive statistics of the research variables were displayed, and the data were presented as ($\bar{X}$ ± *S*). The comparison between two groups was analyzed by chi‐square test or independent sample *t* test, and differences among more than three groups were analyzed by analysis of variance (ANOVA). Each influential factor for cognitive function was examined by Spearman rank correlation analysis or Pearson correlation analysis. Factors with statistical significance were subject to multiple stepwise regression analysis to establish the regression equation. *p* < .05 was considered statistically significant.

## 3 RESULTS

### 3.1 The cognitive function scores of the epileptic patients were lower than the normal reference range

The general information of epileptic patients showed as Table 1. The normal reference ranges of the scales adopted in the current study were formulated as suggested by the previous studies (Folstein et al., 1975; Nasreddine et al., 2005). The results showed that the memory quotient of the epileptic patients was slightly lower than that of the normal subjects, indicating a mild memory decline in the epileptic patients as a whole. The scores of MMSE, DSy, VFT and sequential and backward memory of DSp still fell within the normal range, indicating that these cognitive function tests had a low sensitivity to a mild cognitive impairment. But the scores of MoCA were lower than that of the normal group, indicating that the cognitive function test was sensitive even to a mild cognitive impairment, which is consistent with a previous study in South Korea (Smith, Gildeh, & Holmes, 2007; Table 2).

**Table 2:** The cognitive function of the epileptic patients compared with the normal reference range

	Epileptic group (points)	Normal range (points)
Memory quotient of CMS (*n* = 275)	85.22 ± 19.75	≥90
MMSE (*n* = 70)		
Primary school or below	23.75 ± 5.74	≥21
Junior high school or above	28.18 ± 2.55	≥25
MoCA (*n* = 70)	24.39 ± 4.80	≥26
DSy (*n* = 70)	51.19 ± 15.74	≥45
VFT (*n* = 70)		
Primary school or below	28.75 ± 10.87	≥21
Junior high school or above	38.06 ± 11.04	≥26
Sequential memory of DSp (*n* = 70)	8.31 ± 1.46	≥5
Backward memory of DSp (*n* = 70)	5.5 ± 2.07	≥4

Abbreviations: CMS, clinical memory scale; DSp, digit span test; DSy, digital symbol test; MMSE, mini‐mental state examination; MoCA, Montreal cognitive assessment; VFT, verbal fluency test.

### 3.2 Monadic linear related analysis of possible factors affecting the cognitive function of the epileptic patients

The results revealed that age, educational level, disease duration, seizure frequency, kinds of AEDs, degree of anxiety and depression were, respectively, correlated with the cognitive function of the epileptic patients (Tables 3 and 4).

**Table 3:** Correlation coefficients *r* value of monadic linear related analysis of potential factors affecting the cognitive function of the epileptic patients (*n* = 257)

	Age	Education level	Onset age	Disease duration	Seizure frequency	Seizure types	Kinds of AEDs
Memory quotient	−0.053	0.497**	0.016	−0.116	0.500**	−0.008	−0.540**
Directed memory	−0.166**	0.394**	−0.022	−0.174**	0.358**	−0.033	−0.471**
Associative learning	−0.086	0.473**	−0.009	−0.146*	0.389**	0.078	−0.392**
Free image recall	0.024	0.257**	0.091	−0.142*	0.329**	−0.094	−0.421**
Meaningless graph recognition	0.063	0.355**	0.067	−0.008	0.380**	−0.027	−0.277**
Associative memory of portrait characteristics	0.010	0.469**	0.051	−0.068	0.446**	−0.016	−0.446**

Abbreviation: AEDs, antiepileptic drugs.

a *p < .05; **p < .01.

**Table 4:** Correlation coefficients *r* value of monadic linear related analysis of potential factors affecting the cognitive function of the epileptic patients (*n* = 70)

	Age	Educational level	Onset age	Disease duration	Seizure frequency	Seizure types	Kinds of AEDs	Anxiety	Depression
MMSE	−0.013	0.370**	0.102	−0.107	0.099	−0.096	−0.130	−0.414**	−0.407**
MoCA	−0.058	0.499**	0.008	−0.165	0.299*	−0.214	−0.299*	−0.335**	−0.351**
DSy	−0.027	0.549**	0.114	−0.184	0.412**	−0.205	−0.364**	−0.205	−0.214
VFT	0.070	0.524**	0.130	−0.044	0.246*	−0.204	−0.155	−0.163	−0.175
Sequential memory of DSp	−0.163	0.423**	−0.038	−0.159	0.045	−0.144	−0.162	−0.126	−0.190
Backward memory of DSp	−0.125	0.336**	−0.069	−0.136	0.124	−0.135	−0.218	−0.209	−0.289*

Abbreviations: AEDs, antiepileptic drugs; DSp, digit span test; DSy, digital symbol test; MMSE, mini‐mental state examination; MoCA, Montreal cognitive assessment; VFT, verbal fluency test.

a *p < .05; **p < .01.

### 3.3 Multiple stepwise regression analysis of potential factors affecting the cognitive function of the epileptic patients

The results showed that educational level, seizure frequency, and kinds of AEDs entered the regression equation repeatedly, indicating that these factors can exert great effect on the cognitive function of the epileptic patients. Lower educational level, higher seizure frequency, and more AEDs indicated severer cognitive impairment. In addition, depression severity, disease duration, and age were negatively correlated with the cognitive function. Seizure type was also correlated with the cognitive impairment and general seizure produced the greatest effect on the cognitive function of the epileptic patients (Tables 5 and 6).

**Table 5:** Standardized regression coefficients of multiple stepwise regression analysis of potential factors affecting the cognitive function of the epileptic patients (*n* = 257)

	Age	Educational level	Onset age	Seizure frequency	Seizure types	Disease duration	Kinds of AEDs
Memory quotient	—	0.330**	—	−0.304**	—	—	−0.363**
Directed memory	—	0.258**	—	−0.183**	—	—	−0.353**
Associative learning	—	0.391**	—	−0.222**	—	—	−0.226**
Free image recall	—	0.141*	—	−0.191**	—	—	−0.326**
Meaningless graph recognition	—	0.219**	—	−0.300**	—	—	−0.151*
Associative memory of portrait characteristics	—	0.325**	—	−0.254**	—	—	−0.355**

Abbreviation: AEDs, antiepileptic drugs.

a *p < .05; **p < .01.

**Table 6:** Standardized regression coefficients of multiple stepwise regression analysis of potential factors affecting the cognitive function of the epileptic patients (*n* = 70)

	Age	Educational level	Onset age	Seizure frequency	Seizure types	Disease duration	Kinds of AEDs	Anxiety	Depression
MMSE	—	0.299**	—	—	—	—	—	—	−0.432**
MoCA	−0.229*	0.392**	—	—	—	—	—	—	−0.356**
DSy	—	0.476**	—	−0.287**	−0.187*	−0.257**	—	—	—
VFT	—	0.523**	—	—	—	—	—	—	—
Sequential memory of DSp	—	0.330**	—	—	—	—	—	—	−0.367**
Backward memory of DSp	—	0.292**	—	—	—	—	—	—	−0.242**

Abbreviations: AEDs, antiepileptic drugs; DSp, digit span test; DSy, digital symbol test; MMSE, mini‐mental state examination; MoCA, Montreal cognitive assessment; VFT, verbal fluency test.

a *p < .05; **p < .01.

### 3.4 Effects of educational level, seizure frequency, and kinds of AEDs on the cognitive function of the epileptic patients

According to the educational level of the patients, results showed that except for the score of memory quotient, directed memory, associative memory of portrait characteristics, and MMSE (*p* < .01 or *p* < .05), no significant difference was found in the scores of cognitive function between the primary school group and junior high school group; except for the score of associative learning (*p* < .01), no significant difference in the score of cognitive function was found between the senior high school group and college level or above group; except for the MMSE scores, significant difference in the scores of other cognitive functions was found between the junior high school group and senior high school group (*p* < .01 or *p* < .05) (Tables 7 and 8). These results indicate that an educational level of senior high school or over can offer protective effect on the cognitive function of the epileptic patients.

**Table 7:** Comparison between the scores of CMS across the groups (*n* = 257)

	Primary school group (*n* = 22)	Junior high school group (*n* = 143)	Senior high school group (*n* = 58)	College level or above group (*n* = 34)
Memory quotient	71.06 ± 21.18	78.96 ± 16.78*	97.05 ± 16.55** ^,^ ††	100.55 ± 16.22** ^,^ ††
Directed memory	9.53 ± 7.21	12.90 ± 6.78*	18.09 ± 6.42** ^,^ ††	18.38 ± 6.19^a^, †† ^,^ ††
Associative learning	14.09 ± 6.73	15.60 ± 5.72	21.45 ± 6.37** ^,^ ††	25.06 ± 7.52^a^, ††, ‡‡ ^,^ ††,‡‡
Free image recall	14.97 ± 7.93	16.36 ± 5.83	19.55 ± 5.97^a^, †† ^,^ ††	20.15 ± 6.61** ^,^ ††
Meaningless graph recognition	12.91 ± 6.71	13.76 ± 6.27	18.95 ± 5.23^a^, ^††^	18.04 ± 4.88** ^,^ ††
Associative memory of portrait characteristics	10.59 ± 8.08	13.93 ± 6.40*	19.60 ± 6.60^a^, ††	21.71 ± 5.42** ^,^ ††

Abbreviation: CMS, clinical memory scale.

a *p < .05, **p < .01: mean comparison with the primary school group.

^††^ *p* < .01: mean comparison with the junior high school group.

^‡‡^ *p* < .01: mean comparison with the senior high school group.

**Table 8:** Comparison of other cognitive functions across the groups (*n* = 70)

	Primary school group (*n* = 4)	Junior high school group (*n* = 40)	Senior high school group (*n* = 15)	College level or above group (*n* = 11)
MMSE	23.75 ± 5.74	27.63 ± 3.00**	28.73 ± 1.58**	29.45 ± 0.52**
MoCA	19.75 ± 9.22	23.25 ± 4.54	26.27 ± 3.71* ^,^ †	27.64 ± 1.86** ^,^ ††
DSy	44.00 ± 11.17	44.58 ± 14.00	60.33 ± 15.51* ^,^ ††	65.36 ± 5.30** ^,^ ††
VFT	28.75 ± 10.87	33.58 ± 8.55	43.87 ± 12.61** ^,^ ††	47.18 ± 8.26** ^,^ ††
Sequential memory of DSp	7.50 ± 1.73	7.88 ± 1.52	9.13 ± 1.13* ^,^ ††	9.09 ± 0.54*,††
Backward memory of DSp	5.00 ± 2.16	4.95 ± 2.04	6.20 ± 1.74†	6.73 ± 2.05†

Abbreviations: DSp, digit span test; DSy, digital symbol test; MMSE, mini‐mental state examination; MoCA, Montreal cognitive assessment; VFT, verbal fluency test.

a *p < .05, **p < .01: mean comparison with the primary school group.

^†^ *p* < .05, ^††^ *p* < .01: mean comparison with the junior high school group.

According to the seizure frequencies, the analysis showed that the cognitive function of Group A was significantly different from that of other groups (*p* < .01), indicating that a seizure frequency of less than once a year can provide stronger protection for the cognitive function of the epileptic patients (Table 9).

**Table 9:** Comparison of cognitive function between groups of different seizure frequency (*n* = 257)

	Group A (*n* = 26)	Group B (*n* = 115)	Group C (*n* = 70)	Group D (*n* = 46)
Memory quotient	110.31 ± 10.85	87.99 ± 17.69**	79.81 ± 16.74**, †	72.37 ± 18.26**, †, ‡
Directed memory	21.31 ± 4.15	14.86 ± 6.99**	13.98 ± 7.10**	10.59 ± 6.61**, †, ‡
Associative learning	25.83 ± 5.71	18.63 ± 6.71**	16.41 ± 6.31**	14.66 ± 6.79**, †
Free image recall	22.38 ± 4.72	18.14 ± 6.12**	16.44 ± 5.88**	14.54 ± 6.79**, †
Meaningless graph recognition	21.24 ± 5.30	16.55 ± 6.22**	12.80 ± 5.22**, †	13.31 ± 6.07**, †
Associative memory of portrait characteristics	24.65 ± 4.00	16.70 ± 6.72**	14.54 ± 6.44**	11.30 ± 6.98**, †, ‡

** *p* < .01: mean comparison with Group A.

^†^ *p* < .05, ^††^ *p* < .01: mean comparison with Group B.

^‡^ *p* < .05, ^‡‡^ *p* < .01: mean comparison with Group C.

According to kinds of AEDs taken, results revealed that except for a statistic difference in the free image recall and the associative memory of portrait characteristics (*p* < .05), no significant difference was evident in the other aspects of cognitive function between Group 0 and Group 1; a significant difference in the cognitive function was present between Group 1 and Group 2 or Group 3 (*p* < .01), indicating that single medication may not induce obvious cognitive impairment in epileptic patients (Table 10).

**Table 10:** Comparison of cognitive function of different groups with different kinds of AEDs (*n* = 257)

	Group 0 (*n* = 18)	Group 1 (*n* = 116)	Group 2 (*n* = 105)	Group 3 (*n* = 18)
Memory quotient	100.85 ± 11.77	93.58 ± 17.72	77.66 ± 16.32** ^,^ ††	59.83 ± 14.52** ^,^ †† ^,^ ‡‡
Directed memory	19.78 ± 4.80	17.19 ± 6.31	11.91 ± 6.76** ^,^ ††	7.06 ± 6.11** ^,^ †† ^,^ ‡‡
Associative learning	21.19 ± 5.97	20.70 ± 7.10	15.59 ± 6.11** ^,^ ††	12.11 ± 6.71** ^,^ †† ^,^ ‡
Free image recall	22.56 ± 4.55	19.28 ± 6.02*	15.60 ± 5.90** ^,^ ††	11.50 ± 5.12** ^,^ †† ^,^ ‡‡
Meaningless graphics recognition	16.32 ± 5.21	17.11 ± 6.03	14.37 ± 6.35††	9.81 ± 5.41** ^,^ †† ^,^ ‡‡
Associative memory of portrait characteristics	22.50 ± 5.22	18.64 ± 6.65*	13.33 ± 6.19** ^,^ ††	7.39 ± 5.82** ^,^ †† ^,^ ‡‡

a *p < .05; **p < .01: mean comparison with Group 0.

^††^ *p* < .01: mean comparison with Group 2.

^‡^ *p* < .05; ^‡‡^ *p* < .01: mean comparison with Group 2.

## 4 DISCUSSION

The present study employed a combination of currently widely applied scales to evaluate the cognitive status of epileptic patients. The correlation between their cognitive performance and potential influential factors was analyzed by monadic linear related analysis, multiple stepwise regression analysis, and ANOVA. We found that the general cognitive function of epileptic patients was lower than normal reference range, mainly involving vocabulary difficulty, attention deficit, mental retardation, decline in memory, executive function, and psychomotor speed; and that the effective factors for declined cognitive function were educational level, seizure frequency, seizure types, disease duration, depression, age, and kinds of AEDs.

Of all the effective factors, educational level was found negatively correlated with the cognitive performance of the epileptic patients. The higher the educational level, the lower the cognitive impairment was, and an educational level over senior high school surfaced as a protective factor for the cognitive function of epileptic patients. This finding falls in line with the previous studies (Oyegbile et al., 2004; Taylor & Baker, 2010). One possible explanation lies in the fact that people with a high educational level have more intellectual reserves and stronger ability in understanding and performing different cognitive function tests. Moreover, patients with a high level of education are usually more informed of the disease and better self‐disciplined, so they follow the treatment scheme more strictly, which enhances the treatment effect and reduces cognitive impairment consequently. In social settings, however, many families and schools do not understand epilepsy adequately and reduce the educational opportunities for epileptic patients, coupling the adverse effects on their cognitive function. Therefore, our society should raise public awareness of the disease, show more tolerance toward epileptic patients, and make double efforts to improve their education level.

Seizure frequency has also been documented to be closely associated with the cognitive impairment of epileptic patients. One study suggests that frequent seizures are closely related to the decline in the domains of memory and executive function (Black et al., 2010). Another research reports that the long‐term or repeated seizures can cause or worsen cognitive impairment (Holmes, 2015). Still, another demonstrates that overall cognitive function in epileptic patients declines with the elapsing time and that seizures can increase the risk of progressive decline of cognitive function, namely “epileptic dementia” (De Reuck, Clerck, & Maele, 2006). In the same line, the current study also found that seizure frequency was significantly correlated with the cognitive performance of the epileptic patients. The more frequent the seizures, the more affected the memory and executive functions were. Notably, we found that seizure control for more than 1 year surfaced as a protective factor for improving cognitive function of epileptic patients. Some inconsistent studies suggest that cognitive function is not related to seizure frequency (Miller et al., 2016), which might result from the enrollment of different samples and the employment of different study methods. Epileptic seizure is induced by the excessive synchronous discharge from brain neurons. As a result, hypoxia in the neuronal membrane and electricity failure cause irreversible damage to neurons, and frequent seizures extend the time of abnormal discharge in the gray matter, affecting brain function and resulting in a decline in the cognitive function. Abnormal white matter and gray matter have been documented to be associated with the decline of cognitive function (Vaessen et al., 2012).

For epileptic patients, their cognitive function can also be compromised by the AEDs taken. Studies have reported that epileptic patients suffer decline in verbal memory, visual memory and visual–spatial ability as a result of taking more kinds of AEDs (Piazzini, Turner, et al., 2006) and that the kinds of drugs taken can serve as a predictor of the decline in domains of memory, attention, and executive function (Piazzini, Canevini, Turner, Chifari, & Canger, 2006). The current study also found that the more AEDs epileptic patients took, the more impaired the cognitive function was, including a decline in verbal memory, nonverbal memory, attention, executive function and that patients with monotherapy had the least cognitive impairment. Such a connection can be reasonably interpreted by pharmaceutical facts: most of the AEDs have cognitive side effects, including sleepiness, inattention, insomnia, and dizziness, which accumulate with the mounting number of AEDs; as different kinds of AEDs produce complex pharmacokinetic interactions, the combined neurotoxicity may increase the risk of cognitive impairment when the drugs were metabolized by the liver, even though their blood concentrations fall within the normal range. A recent study has documented that monotherapy can reduce cognitive impairment and each additional drug suggests a decline in cognition (Witt, Elger, & Helmstaedter, 2015). Other studies, however, suggest that cognitive decline is not associated with the kind of AEDs, but with other contributing factors or mechanisms (Piazzini, Turner, et al., 2006), or that cognitive impairment may appear before AEDs treatment (Pulliainen, Kuikka, & Jokelainen, 2000). The inconsistency prompts us to speculate that the kind of AEDs might be a sign of the severity of seizures, that is, epileptic patients using fewer AEDs have lower seizure frequency and mild cognitive impairment. For patients with poor seizure control, more medications may reduce or control attack, thus reducing cognitive impairment, but at the same time, also increase the risk of cognitive impairment. Therefore, a balance should be maintained between seizure control and reducing drug side effects. Studies suggest that optimizing the number of AEDs can reduce or control seizures in patients with drug‐resistant epilepsy and significantly reduce drug‐related side effects, and that it is not necessary to give patients more than three kinds of AEDs, as sometimes changing the combination and adjusting the dose can reduce the side effects and improve the efficiency of epileptic seizure control (Dash, Aggarwal, Joshi, Padma, & Tripathi, 2015).

Recently, depression and anxiety are reported as the most common mental problems of epileptic patients. Whether they are related to cognitive impairment in epilepsy is still under exploration. Studies have reported that depression is closely related to the decline of psychomotor speed (Folstein et al., 1975; Witt et al., 2015), and the high depression scores are associated with declining scores of Dsy (Gul & Ahmad, 2015). Similarly, the current study also showed that the state of anxiety and depression had a side‐effect on the cognitive performance of epileptic patients. After multiple stepwise regression analysis, the influence of anxiety was offset by other factors, but depression remained an important factor for cognitive function. Furthermore, depression and anxiety can also increase the side effects of AEDs (Park, 2013) and determine the final tolerance of AEDs (Petrovski et al., 2010). Available literature also documents that depression can better forecast and decide the quality of life of epileptic patients than epileptic seizures (Luoni et al., 2011; Reilly et al., 2015). Therefore, identifying the nature of depression and anxiety is extremely important for improving the quality of life of epileptic patients due to the two‐way relationship between depression and epilepsy (Kanner, 2012). On the one hand, epileptic patients are more susceptible to depression and anxiety, with 75% higher comorbidity of (Kwon & Park, 2014) and a high prevalence of the two mental health problems (10%–60% and 15%–20%, respectively; Verrotti, Carrozzino, Milioni, Minna, & Fulcheri, 2014); on the other hand, depression patients are more likely to develop epilepsy (Woods & Gruenthal, 2006), with 30%–80% of epileptic patients reporting psychological stress as the trigger of seizures (da Silva Sousa, Lin, Garzon, Sakamoto, & Yacubian, 2005). A case study indicates that special emotional precipitation might induce epileptic seizures (Woods & Gruenthal, 2006). Unfortunately, neuropsychological problems of many epileptic patients are often neglected or untreated (Reilly et al., 2014). Therefore, medical personnel should intervene early before the illness becomes too serious and prescribe for epileptic patients antidepressant treatments and physical exercise, which can effectively reduce anxiety and depression and improve the quality of their life (Arida, Cavalheiro, & Scorza, 2012; Kanner & Balabanov, 2002).

Age is another factor for cognitive impairment of epileptic patients. Various studies have reported that the elderly patients are more prone to obvious impairment in the domains of psychomotor speed (Taylor & Baker, 2010), memory and executive function (Thompson & Duncan, 2005; Witt et al., 2014). In a similar line, this study found that age was negatively correlated with MoCA score after excluding the other factors. Possible explanations may lie in: (a) epileptic patients have normal age‐related cognitive decline, so the older patients have relatively poorer cognitive function; (b) an advancing age means extended disease course, more medications, more drug interactions, increased comorbidity, changing metabolic rate, and increasing susceptibility to cognitive impairment. Therefore, we need to pay more attention to the cognitive function of middle‐aged and elderly epileptic patients and improve the quality of their life in the future.

Over the disease process, disease duration has surfaced as an important factor for cognitive performance of the epileptic patients. Available studies indicate that specific cognitive impairment, such as decline in memory, attention, executive function, naming ability, and verbal fluency, deteriorates with the extending disease course (Taylor & Baker, 2010). The current study also found that disease duration was negatively correlated with the DSy scores. The reason might be that a longer disease duration may induce neuronal damage or loss and produce a cumulative effect, which might lead to abnormal cerebral morphological structure and metabolism and gradually aggravate the cognitive impairment. But some studies suggest that disease duration is not correlated with cognitive function, for cognitive decline of newly diagnosed epileptic patients was not found 5 or 30 years after the diagnosis (Aikia, Salmenpera, Partanen, & Kalviainen, 2001; Griffith et al., 2007). The inconsistency may be explained by the hypothesis (Arida et al., 2012), which maintains that the decline of cognitive function in epileptic patients is waterfall rather than gradual. It argues that neural development interruption and significant decline in cognitive function occur at epilepsy onset, so at a relatively early age, cognitive function of epileptic patients precipitates over the disease course instead of a gradual deterioration. Therefore, there might be different results in the cognitive function tests at different stages of epilepsy.

General seizure type has also been demonstrated to exert significant effect on the cognitive function of epileptic patients (Taylor & Baker, 2010; Thompson & Duncan, 2005). In the present study, general seizure, compared with partial seizure, was more markedly correlated with the cognitive performance of epileptic patients. The explanation might be that the epileptic discharge of the general seizure exists in the bilateral brain, posing greater control difficulty and exerting a greater effect on the cognitive function. There are also studies suggesting cognitive impairment in patients with absence seizure is more significant than other seizure types (Gul & Ahmad, 2015). Different results may be related to different patients with different type conversions so the results might be subject to an overlap effect, or different seizure types may operate through the same mechanism or pathways that lead to inconsistent results.

In summary, educational level, seizure frequency, seizure types, disease duration, depression, age, and kinds of AEDs are significant factors for cognitive impairment of epileptic patients. Early detection of cognitive impairment, intervention and enhancement of cognitive function are worthy of note in daily follow‐up of epileptic patients. Public awareness of epilepsy, better emotional management, optimal medication treatment, and timely cognitive evaluation are of great therapeutic significance for the care of epileptic patients.

## CONFLICT OF INTEREST

The authors declare they have no conflicts of interest according to the subject and matter of the present article.

## Data Availability

The data that support the findings of this study are available from the corresponding author upon reasonable request.
